# Lifestyle differentiation among marine denitrifying microorganisms

**DOI:** 10.1093/ismejo/wrag232

**Published:** 2026-09-04

**Authors:** Xin Sun, Irene H Zhang, Andrew R Babbin, J L Weissman, Emily J Zakem

**Affiliations:** Department of Biology, University of Pennsylvania, Philadelphia, PA 19104, United States; Department of Global Ecology, Carnegie Institution for Science, Stanford, CA 94306, United States; Department of Earth, Atmospheric and Planetary Sciences, Massachusetts Institute of Technology, Cambridge, MA 02139, United States; Department of Earth, Atmospheric and Planetary Sciences, Massachusetts Institute of Technology, Cambridge, MA 02139, United States; Institute for Advanced Computational Science, Stony Brook University, Stony Brook, NY 11794, United States; Department of Ecology and Evolution, Stony Brook University, Stony Brook, NY 11794, United States; Department of Global Ecology, Carnegie Institution for Science, Stanford, CA 94306, United States

**Keywords:** Marine denitrifiers, lifestyles, function, oligotrophy, copiotrophy

## Abstract

Microorganisms carrying out denitrification in marine anoxic zones drive bioavailable nitrogen loss. Sequencing datasets have demonstrated the modularity of denitrification, with most populations having the genetic capability for only a subset of the pathway (NO_3_^−^➔NO_2_^−^➔NO➔N_2_O➔N_2_). Although previous work provided ecological explanations for this diversity among the functional modules, large trait variations exist within each functional module, and this within-module diversity and its biogeochemical implications remain unexplored. Here, we combine genomic data and modeling to explore how metabolic “lifestyle” strategies influence denitrifier community structure. We build a comprehensive genomic database of marine denitrifiers, and identify lifestyle differentiation among denitrifier functional groups. We then extend a mathematical ecosystem model by resolving two microbial functional types for each module representing a metabolic trade-off: a copiotroph, optimized for fast growth, and an oligotroph, optimized for high nutrient affinity. In the model, as the supply of organic matter relative to nitrate increases, the degree of copiotrophy among the community increases and then decreases. This suggests that oligotrophs are associated with either organic-matter- or nitrate-limiting conditions, whereas copiotrophic lifestyles are associated with an intermediate regime. Our model further associates NO_2_^−^ reducers with oligotrophy and NO_3_^−^ reducers with copiotrophy, particularly those producing the greenhouse gas nitrous oxide (N_2_O), linking N_2_O production to substrate-replete conditions, which is consistent with our genome-based lifestyle estimates. Results provide insight into denitrifier ecological niches and thus the biogeochemical conditions that are associated with the production of intermediates, such as N_2_O, improving our understanding of how nitrogen cycling will change in a warming ocean.

## Introduction

Heterotrophic microorganisms rely on organic matter (OM) for energy and biomass production while predominantly using oxygen as the terminal electron acceptor. In environments where oxygen is scarce, such as open-ocean oxygen minimum zones (OMZs), many anaerobic heterotrophs persist by remineralizing OM while using nitrogen instead of oxygen for respiration. Denitrification is one of the most prevalent anaerobic respiration processes in OMZs, in which various nitrogen compounds serve as electron acceptors. The complete denitrification pathway is the stepwise reduction of NO_3_^−^ through NO_2_^−^, NO, N_2_O, and ultimately to N_2_. Microorganisms performing only subsets or “modules” of the full process are much more abundant than those performing the complete pathway in OMZs [1–3]. Here, we refer to all microbes that have the capability to carry out any of these modules as denitrifiers. Modular denitrification pathways are crucial in shaping nutrient conditions in the ocean. The steps that convert NO_3_^−^ or NO_2_^−^ to N_2_O, NO, or N_2_ result in a net loss of bioavailable nitrogen from the ocean [4–7], which is globally biogeochemically relevant because roughly half of the primary production in surface waters is limited by nitrogen.

In recent years, the functional diversity of marine denitrifiers has been explicitly characterized due to advances in genome-resolved metagenomics [2, 3]. Results from both metagenomics and rate measurements show that diverse denitrifiers, including those directly competing for the same resources, coexist in the same water mass. Few populations have the genetic capability to carry out the full denitrification pathway, and certain modules (NO_3_^−^➔NO_2_^−^) are more abundant than others. To understand the coexistence of diverse denitrifiers and their different abundances in distinct environments, recent work developed a theoretical framework [8] to incorporate denitrifier functional groups defined by distinct modules along the denitrification pathway. This previous work constrained biomass yields associated with these functional groups based on the energy produced from their redox reactions and pathway-length penalties. These functional types were then incorporated into an ecosystem model, and the study examined the emergent ecosystem along a gradient in the supply ratio of OM to NO_3_^−^. The model resolved pulses of OM supply, varying in time, to represent the spatial and temporal heterogeneity of resource supply in OMZs from turbulent ocean circulation patterns [9, 10].

This framework provided a mechanistic explanation for the coexistence of diverse denitrifier functional groups in OMZs and reproduced some observed key features of the nitrogen cycle. For example, the results suggested that denitrifiers with shorter steps are more competitive for OM, whereas those with longer steps are more competitive for nitrogen. These results are consistent with the theory of optimal pathway length [11], which predicts that shorter pathways are favored when substrate supply is high, and with fermentation (a shorter pathway) being favored over respiration (a longer pathway) when carbon supply is high [12]. The coexistence of these denitrifiers was attributed to the spatial and temporal heterogeneity of resource supply, such as OM-rich particles and OM-limited free-living conditions present in the same water mass. In addition, the time-varying OM supply, represented as pulses in the model, was critical to capture the dominant N_2_O producing denitrifier functional type (NO_3_^−^➔N_2_O) [13]. This type was favored under an intermediate, co-limitation regime where neither OM nor NO_3_^−^ solely limits growth. This framework is a critical first step toward explaining diversity patterns of denitrifiers.

**Figure 1:**
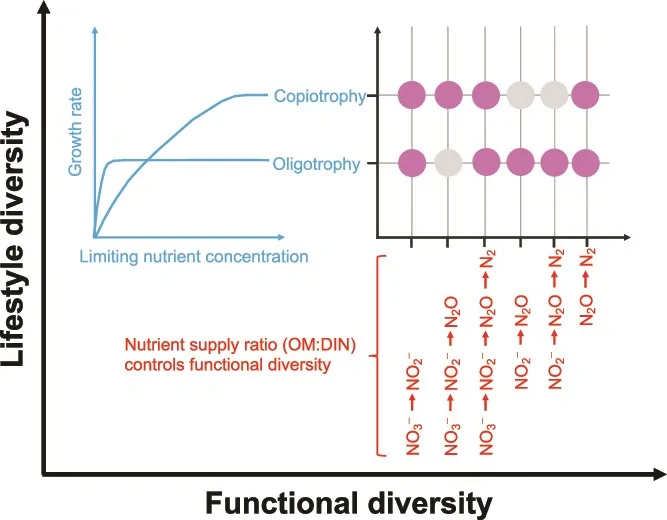
Schematic of denitrifier diversity shaped by two axes: functional diversity defined by different denitrification modules and lifestyle diversity defined by the metabolic rate-affinity trade-off; purple and gray circles indicate denitrifier types that are sustained or competitively excluded by others in our model, respectively.

However, large trait diversity, such as variation in growth rates and biomass yield, is observed within the same denitrifier functional group [14], in addition to the functional diversity. An established way to begin unpacking this within-functional-group diversity is to employ a trait-based approach, where functional types within communities are described by quantitative traits, rather than taxonomic identities [15, 16]. For example, marine aerobic heterotrophs remineralizing OM via aerobic respiration display enormous diversity, and recent modeling work provided insight into their functional biogeography using a traditional classification of bacteria along a copiotrophy-oligotrophy spectrum, often represented as two distinct “lifestyles” [17]. Microbes exhibiting a copiotrophic lifestyle are optimized for relatively rapid growth under nutrient-rich conditions, whereas oligotrophs are optimized for exploiting scarce nutrients [18–21]. The most abundant heterotrophic microbes in the ocean, SAR11 (known as Pelagibacterales), are considered “oligotrophs” [22], defined as such because their high substrate affinity makes them successful with limited OM. In contrast, “copiotrophs,” defined by their potential for rapid growth when substrate is abundant, are often sustained by a “boom and bust” lifestyle driven by a time-varying supply of nutrients due to blooms in productivity. A common mechanism underlying this lifestyle difference is a rate-affinity trade-off, arising from the limited resources that a cell can allocate to different cellular machineries. That is, evolution toward a larger maximum substrate uptake rate may come at the cost of reducing the ability to invest in other fitness strategies such as high substrate affinity (Fig. 1). A classic example is the enzyme Rubisco, the most abundant enzyme on Earth, whose maximum reaction rate and substrate affinity are strongly negatively correlated [23].

Because denitrifiers consist of phylogenetically diverse microorganisms, a denitrifier functional group associated with one module of the denitrification pathway may include microbes with a range of different lifestyles, and certain functional types might overall preferentially exhibit one lifestyle over the other (Fig. 1). This connection between metabolic function and lifestyle is important because it can inform the environmental conditions under which certain denitrification pathways are stimulated and particular intermediates accumulate, linking physiology and metabolic strategy to biogeochemical outcomes. If a functional group is associated with an oligotrophic lifestyle with respect to a particular nutrient, the denitrification pathway it carries out will be favored when that nutrient is depleted. In contrast, pathways linked to copiotrophic denitrifiers are more likely to be stimulated under nutrient-replete conditions or following a sudden supply of their essential resources. When different steps in the pathway are associated with distinct lifestyles, shifts in nutrient conditions can decouple intermediate production from consumption. For example, if N_2_O producers are primarily copiotrophs whereas N_2_O consumers are oligotrophs, conditions with dynamic and/or abundant supply of OM could favor the net production of N_2_O.

To assess lifestyle patterns of marine denitrifiers, we built a comprehensive marine denitrifier genome database and estimated the lifestyle of different denitrifier functional groups using a recently developed genome-based copiotrophy index [17]. To understand observed denitrifier lifestyle patterns and their connection with denitrification functions, we analyzed denitrifier life strategies using a theoretical ecosystem model. Current definitions of copiotrophy and oligotrophy largely consider a single limiting resource (typically OM for heterotrophs). Here we ask how these lifestyles emerge when microbial growth is governed simultaneously by multiple essential resources. We extended our theoretical model [8] to resolve the rate-affinity trade-off axis by expanding each previous denitrifier functional group into two lifestyle types: a copiotrophic type and an oligotrophic type. We used the model to explore nutrient niches of denitrifiers associated with different functions and lifestyles, assess whether particular denitrifier functional groups exhibit lifestyle preferences, and evaluate overall patterns of oligotrophy versus copiotrophy in the denitrifier community. As in previous work, we examined the niches that emerge from ecological interactions among the functional types and their environment along a gradient of the supply ratio of the two key nutrients, OM and NO_3_^−^. Through the combination of observations and modeling, we aimed to unpack the heterogeneity of denitrification pathways in different environments by mechanistically understanding the niche of different microbial types associated with those pathways.

## Materials and methods

### Genome-based estimation of copiotrophy index for marine denitrifiers

We compiled a comprehensive genome database of marine denitrifiers by including 373 isolate genomes, 104 metagenome-assembled genomes (MAGs), and 15 single-cell amplified genomes (SAGs) derived from the global ocean. Isolate genomes were obtained from the National Center for Biotechnology Information (NCBI) database and screened for marine origin. MAGs and SAGs were collected from global ocean metagenomes, with particular emphasis on datasets from OMZs, which were often underrepresented in global genome collections [3]. We identified denitrifiers based on the presence of at least one denitrification gene. Genomes and draft genomes were annotated using PROKKA v1.14.6 [24] against the High-quality Automated and Manual Annotation of Proteins (HAMAP) [25] and Protein families (Pfam) [26] databases. We minimized the risk of protein misannotation while maximizing the retrieval of non-canonical genes or distant homologs using Hidden Markov Models (HMMs) for denitrification. Nitrite-oxidizing bacteria and ammonia-oxidizing archaea and bacteria were removed from the database as they are not known to perform denitrification despite encoding denitrification genes. The HMM for denitrification genes was built following the previous study [3]. We then classified these denitrifiers into six functional groups based on the specific combinations of denitrification genes present in each genome (Supplementary Fig. S1), following the framework used in our model.

To assess their lifestyles, we applied a recently developed algorithm [17, 27] to estimate the degree of copiotrophy of each marine denitrifier in our database based on its genome, MAG or SAG. In the previous study, this degree was defined as the copiotrophy index and was a function of three components associated with copiotrophy: codon usage bias (dCUB) [28], number of carbohydrate active enzymes (nCAZy) [29], and the total number of genes (nGenes). For denitrifiers possessing the same denitrification genes (corresponding to the same functional group in the model) at a given OMZ depth, we calculated a weighted copiotrophy index *I_w_* by accounting for the relative abundance (RA) of each MAG or SAG:


(1) \begin{equation*} {I}_w=\frac{\sum_1^j{I}_i\times{RA}_i}{\sum_1^j{RA}_i} \end{equation*}

Here, *I_i_* is the copiotrophy index of an individual MAG or SAG, *i*. *RA_i_* is its RA at the given OMZ depth. RA of each MAG or SAG was estimated with CoverM using the flags minimap2-sr—min-read-aligned-percent 50—min-read-percent-identity 0.95—min-covered-fraction 0 (https://github.com/wwood/CoverM), following a previous study [3]. *j* is the total number of MAGs or SAGs within a denitrifier functional group at that OMZ depth. We performed this calculation across 16 OMZ depths spanning five stations in the Eastern Tropical North Pacific and Arabian Sea, with three to four depths sampled per station (Supplementary Table S1).

### Representation of lifestyles in a redox-based modeling framework

We extended our recently developed redox-based modeling framework [8] by introducing lifestyle as a new axis governing denitrifier diversity. In the original framework, six functional groups of heterotrophic denitrifiers were described by their unique denitrification modules. The microbial redox reaction associated with each denitrification module is listed in the Supplementary Methods. These pathways are NO_3_^−^➔NO_2_^−^, NO_3_^−^➔NO_2_^−^➔N_2_O, NO_3_^−^➔NO_2_^−^➔N_2_O➔N_2_, NO_2_^−^➔N_2_O, NO_2_^−^➔N_2_O➔N_2_, and N_2_O➔N_2_ (Fig. 1).

In the model, each denitrifier functional type is assumed to carry out its encoded denitrification pathway without exchanging intermediates with the external environment. For example, NO_3_^−^➔NO_2_^−^➔N_2_O denitrifiers are assumed to reduce NO_3_^−^ all the way to N_2_O, rather than releasing or taking up NO_2_^−^. This assumption is supported by our previous work suggesting that most NO_3_^−^➔NO_2_^−^➔N_2_O denitrifiers in marine OMZs are unlikely to exchange nitrite [13, 30]. However, other multistep denitrifiers may release or consume intermediate nitrogen species. Under such conditions, a metabolically flexible organism could effectively occupy different functional roles represented in the model over time, depending on whether intermediates are retained intracellularly or exchanged with the external environment. Explicitly representing such metabolic flexibility and intermediate exchange may be important to understand denitrifier niche partitioning and nitrogen concentration [31], and therefore represents an important direction for future work.

The growth rate (*μ*) of each functional group is governed by its traits (maximum uptake rate, *V_max_*, half-saturation constant, *K*, and biomass yield, *y*) and the limiting nutrient concentration, *R* (one of the two non-substitutable nutrients), following:


(2) \begin{equation*} \mu =\min \left[{y}_{R1}{V}_{{\mathit{\max}}_{R1}}\frac{R1}{R1+{K}_{R1}},{y}_{R2}{V}_{{\mathit{\max}}_{R2}}\frac{R2}{R2+{K}_{R2}}\right] \end{equation*}

The competitiveness of a functional group for a limiting nutrient in a steady-state environment (i.e. when there are no time-varying bloom-like conditions) can be evaluated by its subsistence concentration, R^*^ [32]. R^*^ is the concentration of a nutrient that sustains a functional group when its growth rate equals its loss rate (L) at steady state:


(3) \begin{equation*} {R}^{\ast }=\frac{K_RL}{y_R{V}_{{\mathit{\max}}_R}-L} \end{equation*}

A smaller R^*^ of a nutrient indicates greater competitiveness of a functional group when that nutrient is limiting. In this original framework, the substrate uptake kinetic parameters were kept the same for all types using the same substrate, and only biomass yields varied, reflecting the energetic differences in the underlying redox chemistry. Thus, the six denitrifier groups differed in their denitrification modules and associated biomass yields, but not in their substrate uptake strategies.

In our current study, we extended the original framework with six denitrifier functional groups by introducing lifestyle differentiation within each functional group. In particular, each of the six denitrifier functional groups was composed of two types, one copiotrophic type and one oligotrophic type (Fig. 1), to resolve the lifestyle spectrum along the rate-affinity trade-off axis, characterized by different *V_max_* and *K* values (Supplementary Methods). This lifestyle axis expanded the types of denitrifiers in the model from 6 to 12.

For the newly introduced lifestyle axis, the copiotrophic type had larger values of maximum growth rate, *K* and R^*^ for both non-substitutable nutrients (OM and nitrogen) compared to the oligotrophic type (Supplementary Fig. S2). Here, we assume that copiotrophic and oligotrophic strategies are organism-wide traits, and so we assume that the trade-off between rate and affinity is with respect to both OM and nitrogen uptake simultaneously. This is in contrast to a hypothetical substrate-specific definition of copiotrophy, in which an organism may be “copiotrophic” for OM but “oligotrophic” for nitrogen, which conflicts with our understanding of copiotrophy as a holistic characteristic of the organism. Though a trade-off among acquisition traits for different resources is realistic [33], we assume that this latter trade-off is beyond the scope of copiotrophy. We make this assumption because traits commonly associated with fast-growing copiotrophs, including larger genome sizes and higher ribosomal RNA (rRNA) operon copy numbers, are not resource specific [18, 34, 35]. However, most previous studies of heterotrophic copiotrophs have focused on OM acquisition [17, 36], with much less attention paid to nitrogen. Because of this uncertainty, we also performed a supplemental model experiment in which copiotrophic and oligotrophic strategies differed only in OM uptake strategies whereas nitrogen uptake traits were held constant (Supplementary Fig. S3).

We initialized these 12 denitrifier types with equal abundances in a virtual chemostat, and let the system evolve while supplying constant NO_3_^−^ and pulses of OM (concentration is expressed per unit nitrogen) following a previous protocol [27, 35, 36] to mimic an anoxic OMZ layer. We varied the ratio of average pulsed OM supply to constant NO_3_^−^ supply to the virtual chemostat, and analyzed the results under each supply ratio once microbial biomass and resource concentrations reached a dynamic, time-varying equilibrium state in which they oscillated in a repeated pattern over time (Supplementary Fig. S4). Microbial biomass and resource concentrations at this time-varying equilibrium were averaged over the oscillation cycles for subsequent analyses.

## Results and discussion

### Genomic evidence for lifestyle differentiation among denitrifiers

**Figure 2:**
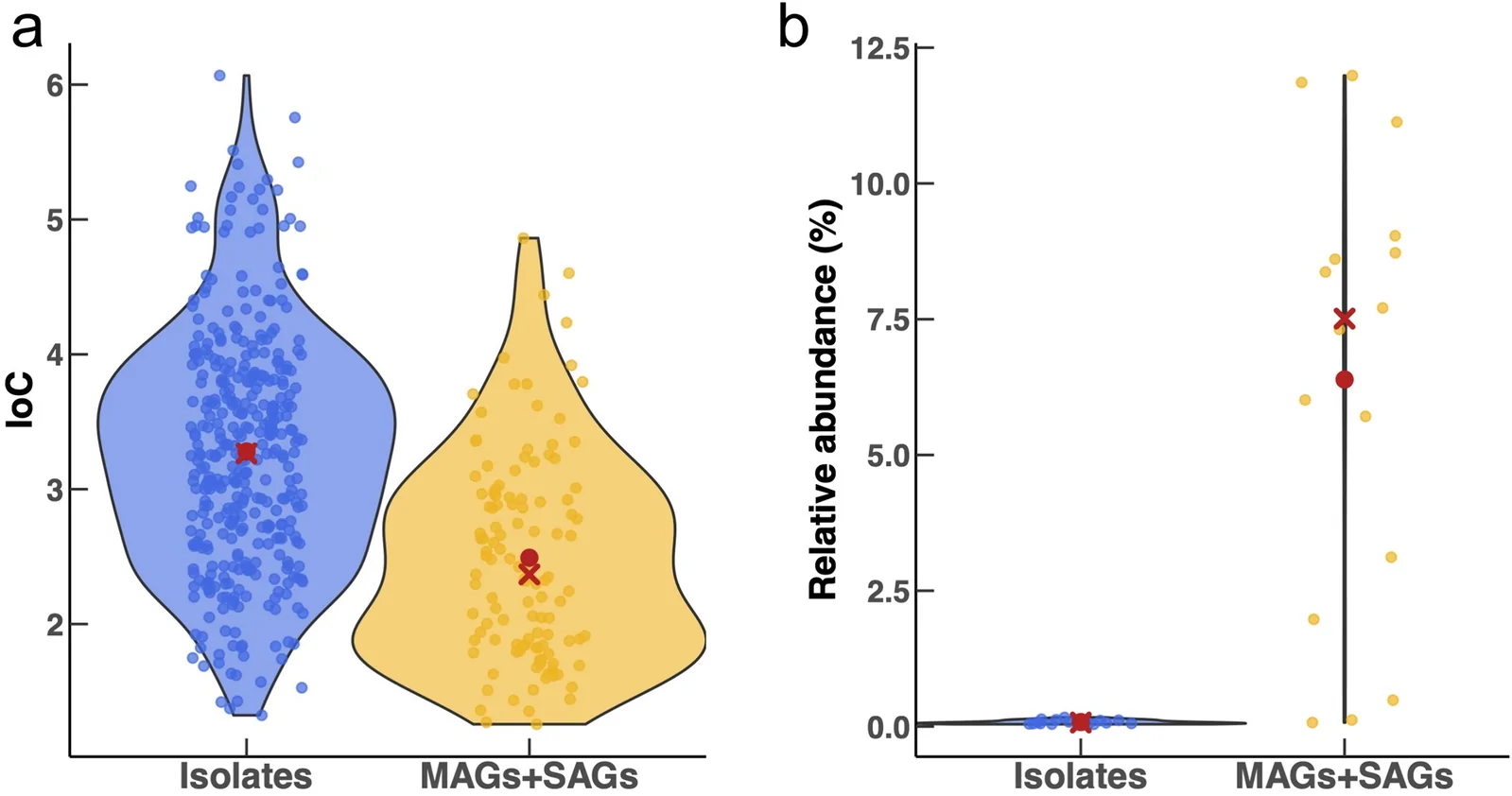
Comparison of the copiotrophy index (a) and RA (b) between isolate genomes and MAGs and SAGs; red cross and circle represent the median and the mean of a violin plot, respectively.

To assess denitrifier lifestyle patterns, we examined a newly constructed dataset of marine denitrifiers and estimated their degree of copiotrophy, using a previously published genome-based copiotrophy index algorithm [17, 27]. First, we find that genomes of denitrifier isolates exhibit significantly higher copiotrophy index values compared to MAGs and SAGs (Fig. 2a), likely reflecting cultivation bias whereby nutrient-rich laboratory conditions select for copiotrophic traits [28, 37, 38]. This result implies that isolate genomes, despite their completeness, are less representative than MAGs and SAGs of the *in situ* marine denitrifier communities, which is further verified by the RA analysis. Even though the total number of isolate genomes (373) is much larger than that of MAGs and SAGs (119), the total RA of all these isolates at any of the 16 depths across five OMZ stations (Supplementary Table S1) is below 0.2%, but the total RA of denitrifier MAGs and SAGs reaches up to 12% (Fig. 2b). This again shows that isolates poorly represent *in situ* denitrifier communities in OMZs. Therefore, we focus on MAGs and SAGs for the following analysis.

**Figure 3:**
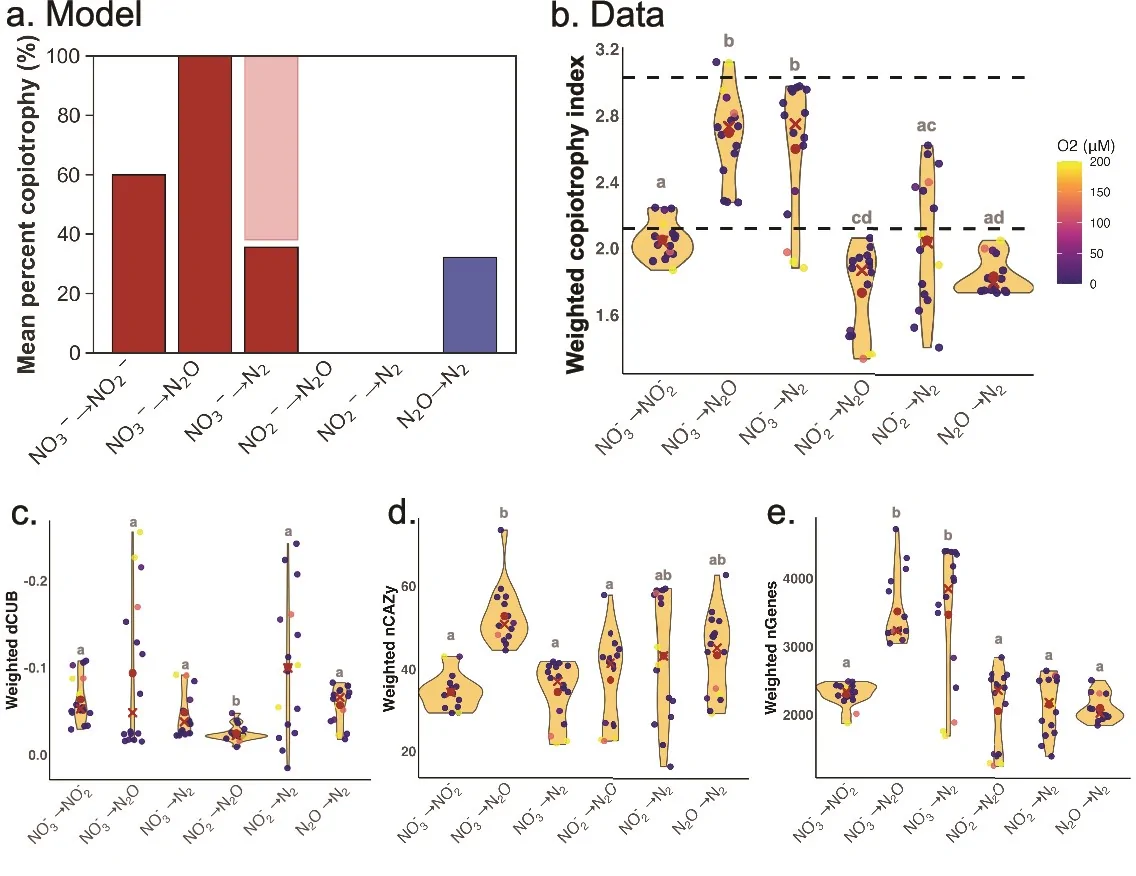
Lifestyle of different denitrifier functional groups estimated by the model and calculated from metagenomes; (a) modeled mean percent copiotrophy of different denitrifier functional groups computed by averaging percent copiotrophy across modeled OM:NO_3_^−^ supply values; for the NO_3_^−^➔N_2_ functional group, the mean percent copiotrophy varies with the selected range of OM:NO_3_^−^: here we vary this range from the minimum value at which at least one other denitrifier functional group is present (solid red bar), to the maximum value considered in our model (transparent red bar); (b) relative-abundance weighted copiotrophy index of different denitrifier functional groups calculated from metagenomes; (c–e) the three components of the copiotrophy index: relative-abundance weighted dCUB [28], nCAZy [29], and nGenes; violin plots that do not share a letter are significantly different from each other in b–e; each datapoint in b–e is a RA-weighted index at a certain OMZ depth; color of the datapoint indicates the *in situ* oxygen concentration of the depth; red cross and circle represent the median and the mean of a violin plot, respectively; black dashed lines in b are thresholds between oligotrophs, slow copiotrophs, and fast copiotrophs defined in a previous study [17].

The resulting copiotrophy indices of NO_3_^−^ reducers are on average higher than those of NO_2_^−^ reducers (Fig. 3b). The differences between most pairs of one NO_3_^−^ reducer type and one NO_2_^−^ reducer type are statistically significant, with the exception of the difference between NO_3_^−^➔NO_2_^−^ denitrifiers and NO_2_^−^➔N_2_ denitrifiers. In addition, the copiotrophy indices of NO_3_^−^➔N_2_O denitrifiers are significantly higher than those of most other denitrifier functional groups, with the exception of complete denitrifiers (NO_3_^−^➔N_2_) with insignificantly smaller copiotrophy indices. Furthermore, the NO_3_^−^➔N_2_O denitrifier type is also the only type that has all samples classified as copiotroph-dominated according to established thresholds [17]. Analysis of individual components (dCUB, nCAZy, and nGenes) of copiotrophy index for MAGs and SAGs (Fig. 3c–e) reveals that NO_3_^−^➔N_2_O denitrifiers possess the highest nCAZy values among all functional groups (Fig. 3d), reinforcing their ecological strategy of efficiently exploiting sudden pulses of OM.

**Figure 4:**
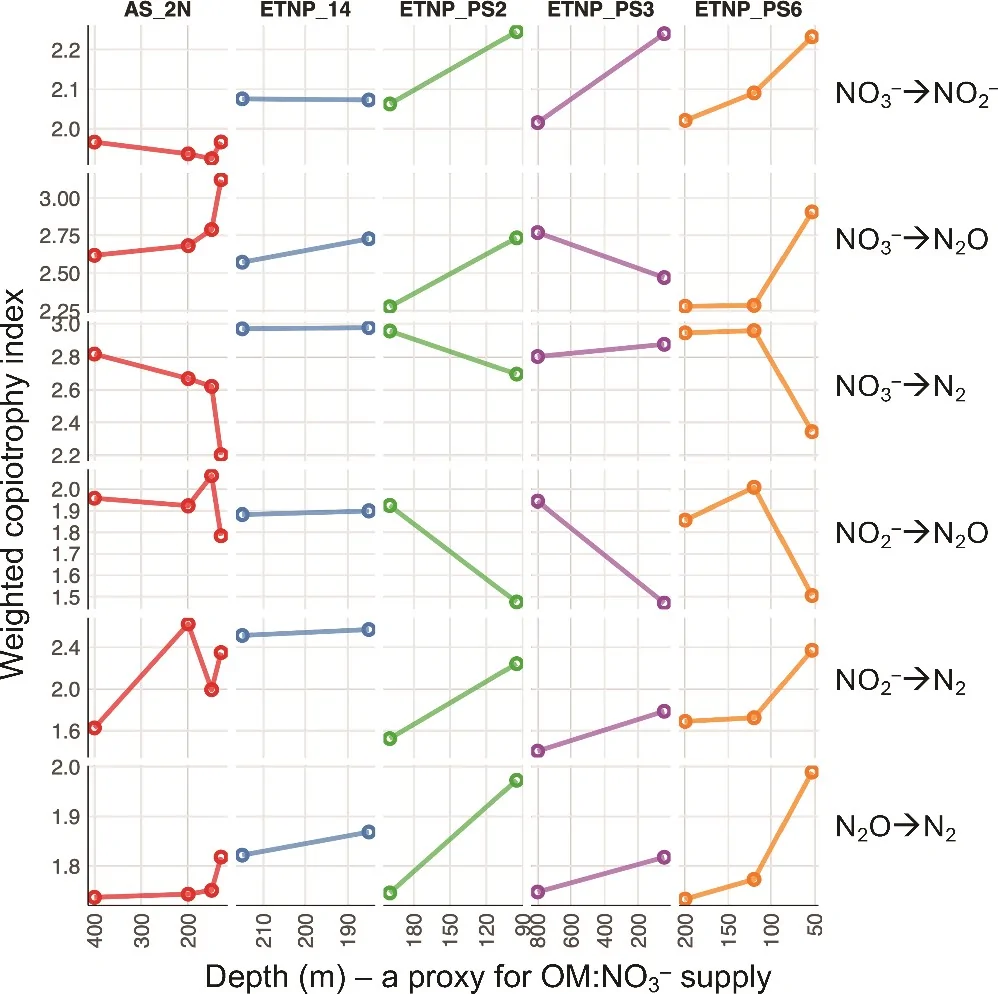
The change of weighted copiotrophy index of each denitrifier functional group with decreasing depth at different stations calculated from metagenomes; depths with oxygen concentration larger than 50 μM are excluded from this figure because they contain very few denitrifiers.

The copiotrophy indices of the NO_3_^−^➔NO_2_^−^ and NO_3_^−^➔N_2_ denitrifier types exhibit opposite responses to increasing OM availability (Fig. 4). We assume that OM availability decreases with increasing depth based on the well-established, widely observed attenuation of OM flux described by the Martin curve [39]. At most OMZ stations, the copiotrophy index of the NO_3_^−^➔NO_2_^−^ denitrifiers increases as depth gets shallower (more OM supply), but that of NO_3_^−^➔N_2_ denitrifiers generally decreases (Fig. 4).

### Model predictions consistent with genomic estimates

**Figure 5:**
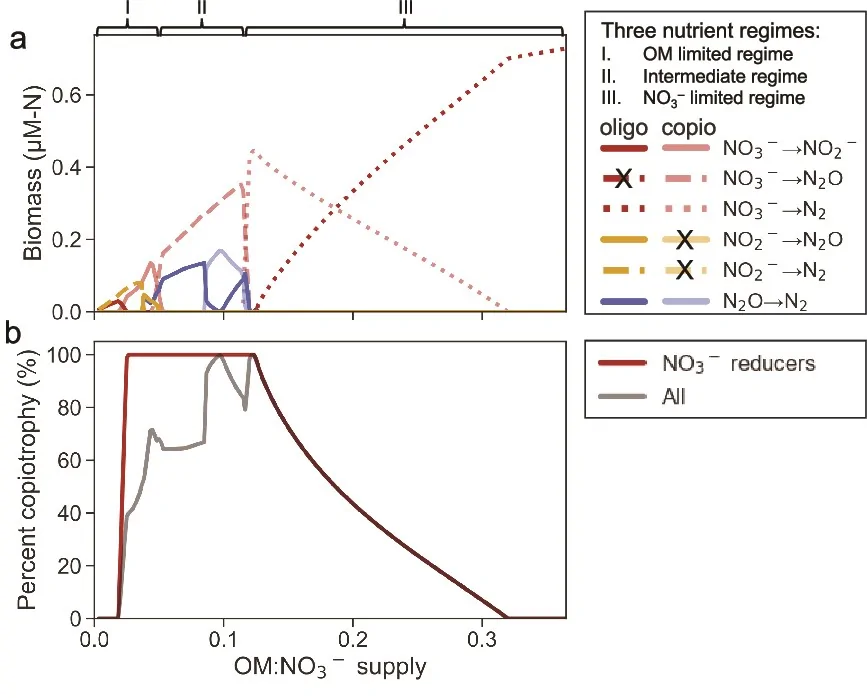
Resulting modeled biomasses and percent copiotrophy of different denitrifiers along an OM:NO_3_^−^ supply gradient; OM concentration is expressed per unit nitrogen; (a) biomasses of different denitrifier lifestyle types and functional groups along an OM:NO_3_^−^ supply gradient; line colors correspond to the inorganic nitrogen substrate consumed by each denitrifier type, line types correspond to step length, and transparency of a line indicates the lifestyle; microbial groups that are not sustained across the tested nutrient conditions are indicated by a black cross in the legend; (b) percent copiotrophy (%) for nitrate reducers and for all denitrifiers calculated as the fraction of biomass attributed to copiotrophs relative to total biomass (copiotrophs + oligotrophs), expressed as a percentage.

To investigate the mechanisms giving rise to these genomic patterns, we incorporated lifestyle differentiation into our ecosystem model. We extended the denitrifier functional group model [38] by incorporating a “lifestyle” (oligotroph-copiotroph) axis by splitting each functional group into two lifestyle types: one copiotrophic type and one oligotrophic type (Fig. 1). We examined their biomass across a range of OM:NO_3_^−^ supply under dynamic (time-varying) equilibrium states where OM was supplied in pulses. This intermittent supply of OM reflects its heterogeneous availability in the ocean [39], and makes the presence of both copiotrophs and oligotrophs possible [40]. Model results show that considering lifestyles in addition to functions (Fig. 5) does not change the functional succession of the dominant denitrifier modules along the OM:NO_3_^−^ supply ratio gradient that was predicted by the previous modeling study [39]. As the OM:NO_3_^−^ supply increases, the denitrifier community transitions from single-step NO_3_^−^ reducers favored under the OM-limited regime, to two-step reducers under an intermediate nutrient regime where neither substrate is limiting, and finally to three-step NO_3_^−^ reducers under the NO_3_^−^-limited regime (Fig. 5a). The mechanism of this succession remains the same, which is that shorter steps are more competitive for limited OM whereas longer steps are favored when inorganic N is limiting. This is consistent with the theory of optimal pathway length, which predicts shorter pathways to be favored when substrate supply is high [11]. However, with lifestyle incorporated, each functional group now has the potential to exhibit two lifestyles.

Different lifestyles are selected along the OM:NO_3_^−^ supply gradient across the three nutrient regimes (Fig. 5a). Under the OM-limited regime, the single-step NO_3_^−^➔NO_2_^−^ functional group initially exhibits an oligotrophic lifestyle but shifts toward a copiotrophic lifestyle as OM:NO_3_^−^ supply increases. The NO_2_^−^ produced by this functional group supports NO_2_^−^ reducers that consistently exhibit an oligotrophic lifestyle throughout this regime. As OM:NO_3_^−^ supply further increases into the intermediate nutrient regime, where neither substrate is limiting, the NO_3_^−^➔N_2_O functional group is exclusively copiotrophic, and it provides N_2_O to N_2_O reducers, which exhibit both lifestyles. Under the NO_3_^−^-limited regime, complete denitrifiers (NO_3_^−^➔N_2_) dominate and shift in the opposite direction from single-step NO_3_^−^➔NO_2_^−^ denitrifiers. They transition from a copiotrophic to an oligotrophic lifestyle as the OM:NO_3_^−^ supply increases. Even though OM is the apparent limiting nutrient in the bulk OMZ environment, increasing OM supply, which results in the increase of OM:NO_3_^−^ supply, does not always promote copiotrophic denitrifier types.

These patterns are consistent with our genomic results, which show that the copiotrophy indices of NO_3_^−^ reducers are on average higher than those of NO_2_^−^ reducers, with NO_3_^−^➔N_2_O denitrifiers having the highest average copiotrophy index among all types (Fig. 3b). Genomic analysis also shows that the responses of the copiotrophy indices of the NO_3_^−^➔NO_2_^−^ and NO_3_^−^➔N_2_ denitrifiers to increasing OM supply are opposite (Fig. 4).

### Ecological mechanisms explain observed genomic patterns

In the model, the percent copiotrophy of all denitrifiers first increases and then decreases as OM:NO_3_^−^ supply increases (Fig. 5b). This is mainly driven by the increase of the percent copiotrophy of NO_3_^−^➔NO_2_^−^ denitrifiers as OM:NO_3_^−^ supply increases but the decrease of the percent copiotrophy of NO_3_^−^➔N_2_ denitrifiers (Fig. 5a and b). This seemingly opposite response of the two NO_3_^−^ reducers can be explained when we consider their growth structured by two nutrients, OM and NO_3_^−^. As shown in our previous study, shorter denitrification modules, here NO_3_^−^➔NO_2_^−^, are more competitive when OM is more limiting than NO_3_^−^ [40, 41]. When OM is the primary limiting resource, the growth of both oligotrophic and copiotrophic types within this functional group is controlled by OM uptake kinetics. Thus, as OM supply rate increases, NO_3_^−^➔NO_2_^−^ denitrifiers shift from an oligotrophic type to a copiotrophic type, because copiotrophs achieve higher growth rates under higher OM supply. Analogously, at high OM:NO_3_^−^ ratios where NO_3_^−^ remains more limiting than OM, the growth of oligotrophic and copiotrophic NO_3_^−^➔N_2_ denitrifiers is set by NO_3_^−^ availability instead of OM. Thus, when OM:NO_3_^−^ supply increases as NO_3_^−^ becomes more limiting, NO_3_^−^➔N_2_ denitrifiers shift from a copiotrophic lifestyle to an oligotrophic lifestyle. This suggests that copiotrophy, as an organism-wide trait, may not be accurately interpreted when considering only OM-limited growth (even though OM is the apparently limiting nutrient in the bulk environment) (Fig. 5). Instead, the model illustrates how different patterns of microbial lifestyle emerge when considering how multiple essential resources limit growth. Thus, the model suggests that maximum community-level copiotrophy occurs not when one resource is abundant, but when neither essential resource is strongly limiting. This extends the traditional single-resource interpretation of copiotrophy, linking copiotrophy to organic carbon availability alone, to the explicit consideration of multiple essential resources.

In contrast, when only OM and not nitrogen acquisition traits were associated with copiotrophy in our supplemental model experiment (Supplementary Fig. S3), copiotrophy increased monotonically with the OM:NO_3_^−^ ratio because lifestyle differences were determined entirely by OM availability, rather than peaking at intermediate OM:NO_3_^−^ ratios as in the default model. Unlike the default model, this monotonic increase was not qualitatively consistent with our genomic analysis. This reinforces our assumption that copiotrophic vs. oligotrophic strategies relate to both OM and nitrogen acquisition in a coupled manner, reflecting our understanding of copiotrophy as an organism-wide rather than resource-specific trait. Nevertheless, future experimental work is needed to verify this assumption.

While modeled NO_3_^−^➔NO_2_^−^ and NO_3_^−^➔N_2_ denitrifiers exhibit an oligotrophic lifestyle when their limiting nutrients become more available as described above, NO_3_^−^➔N_2_O denitrifiers consistently exhibit a copiotrophic lifestyle across various nutrient supply ratios tested in our model (Fig. 5a). This is consistent with their ecological niche in the model—an intermediate regime where neither essential substrate is limiting, and nutrient supply is dynamic—supporting a copiotrophic lifestyle that evolves to grow rapidly when a large pulse of nutrient becomes available. In marine OMZs, such intermediate nutrient conditions are expected to occur near the upper oxycline, where episodic OM inputs coincide with abundant nitrate. This region is also where the highest rates of NO_3_^−^➔N_2_O have been reported [13, 30]. This finding may also help explain observations from other systems. In wastewater treatment plants, high N_2_O production from denitrification is associated with transient substrate availability instead of high organic carbon supply [41, 42]. In soils, hot moments of N_2_O emissions often follow dynamic events such as the application of fertilization, conditions that temporarily relieve resource limitation and favor rapid microbial growth, consistent with a copiotrophic lifestyle [43–45]. These systems may share a common ecological mechanism: intermediate nutrient regimes and dynamically changing environments preferentially favor copiotrophic N_2_O producers.

In contrast to the modeled NO_3_^−^ reducers, which all exhibit a copiotrophic lifestyle under certain conditions, the NO_2_^−^ reducers exclusively prefer an oligotrophic lifestyle (Fig. 5). This is due to the limited supply of NO_2_^−^ for the NO_2_^−^ reducers from NO_3_^−^➔NO_2_^−^ denitrifiers. As longer steps are more competitive when inorganic nitrogen is limiting, the longer, two-step NO_2_^−^ reducers (NO_2_^−^➔N_2_ denitrifiers) are present under most conditions as opposed to the shorter, one-step NO_2_^−^ reducers (NO_2_^−^➔N_2_O denitrifiers). However, NO_2_^−^➔N_2_O denitrifiers are present in a very narrow nutrient niche where copiotrophic NO_3_^−^➔NO_2_^−^ denitrifiers produce NO_2_^−^ faster than their oligotrophic counterparts and NO_2_^−^ accumulates to more than eight micromolar. This functional group cannot be sustained when the amount of accumulated NO_2_^−^ decreases under different frequencies of OM supply (Supplementary Fig. S5). This is consistent with observations of NO_2_^−^➔N_2_O metabolism restricted to only anoxic OMZ waters where NO_2_^−^ builds up [46]. In the previous model lacking lifestyle differentiation, NO_2_^−^➔N_2_O denitrifiers were entirely excluded due to a competitive disadvantage under NO_2_^−^ limitation [8, 47]. The inclusion of lifestyles now allows for the emergence of this rare but ecologically significant group (Fig. 5a). Together, these modeling results provide a mechanistic explanation for the genomic observation that NO_2_^−^ reducers exhibit more oligotrophic lifestyles than NO_3_^−^ reducers (Fig. 3b).

Representing the time-varying supply of resources in OMZs, here modeled as pulses of OM, has been shown to be necessary to reproduce key nitrogen dynamics and features [8, 47], and to support copiotrophic and oligotrophic lifestyles within the same functional group [40]. As the temporal heterogeneity of OM supply in OMZs is derived from processes operating across different time scales, we explore how varying the frequency of OM pulses affects the degree to which copiotrophy is selected. In addition to our default pulse interval (every 50 days), which assumes OM pulses caused by physical processes such as eddies occurring on weekly to monthly timescales, we test scenarios with varying pulse intervals ranging from infrequent (100 days) to highly frequent (5 days) (Supplementary Fig. S5). When OM pulses become very frequent (every 5 days, faster than the slowest microbial turnover time of 5.3 days, calculated from 1/maximum growth rate) and individual pulses are small, resource concentrations fluctuate over a narrower range. This reduces the advantage of pulse-adapted copiotrophs and results in the exclusive presence of oligotrophs. The resulting patterns under other scenarios are qualitatively robust across different pulse frequencies. Copiotrophy consistently peaks at the intermediate OM:NO_3_^−^ supply, and NO_3_^−^ reducers remain consistently more copiotrophic than NO_2_^−^ reducers.

## Conclusions

In this study, we uncover denitrifier lifestyle diversity by estimating the degree of copiotrophy among a comprehensive genomic dataset of denitrifiers. We then explore the potential mechanisms underpinning this diversity within the same functional group by integrating lifestyle strategies using trade-offs between growth rate and substrate affinity in a theoretical model. Results show how functions and lifestyles jointly determine denitrifier niche differentiation along a nutrient supply gradient. These results also suggest that genome-derived lifestyle metrics, such as the copiotrophy index, can provide potential inference for ecological dynamics and help interpret biogeochemical responses.

The genomic analyses and model results show consistent patterns of lifestyle differentiation among denitrifier functional groups (Fig. 3a and b). These results suggest that NO_3_^−^ reducers are generally more copiotrophic than NO_2_^−^ reducers, with NO_3_^−^➔N_2_O denitrifiers, the dominant N_2_O producers in OMZs [13], being the most copiotrophic functional group. This association between functions and lifestyles enables the prediction of how different nutrient dynamics in a changing ocean promote specific denitrification modules and their associated harmful intermediates. Specifically, our model shows that the intermediate nutrient regime preferentially stimulates fast-growing, pulse-adapted copiotrophic N_2_O producers over the consumers, potentially leading to higher N_2_O emissions. This nutrient regime resembles conditions near the base of the upper oxycline in OMZs with episodic OM inputs and non-limiting NO_3_^−^, where N_2_O concentration maxima are observed [13, 30, 46].

Our model results also highlight that the fraction of the overall denitrifier community exhibiting copiotrophic lifestyles peaks at the intermediate nutrient regime (Fig. 5b), where neither OM nor NO_3_^−^ is limiting. Lifestyles are defined with respect to the resource limiting microbial growth under a given condition. Thus, environments with high OM availability, such as the highly productive coastal ocean or OM-rich particles, may not necessarily favor copiotrophic denitrifiers. These results underscore the need to incorporate multiple essential nutrients of microbial groups when predicting responses of microbial functions to changing nutrient conditions. Together, these findings reveal that diversity within denitrifier functional groups can emerge along a lifestyle axis rooted in trade-offs between growth rate and substrate affinity and highlight the previously underappreciated connection between lifestyles and functions.

## Code availability

All codes for the 0-D model in this study and figure generation are deposited in GitHub (https://github.com/xinsun12/Lifestyle-Differentiation-Among-Marine-Denitrifying-Microorganisms).

## Supplementary Material

1_Supplement_revised_clean_wrag232

## Data Availability

The data generated in this study have been deposited in a GitHub repository (https://github.com/xinsun12/Lifestyle-Differentiation-Among-Marine-Denitrifying-Microorganisms) as CSV files.
