# Supplementary material for: Lifestyle differentiation among marine denitrifying microorganisms
Source: ISME J. 2026 Sep 4;20(1):wrag232. doi: 10.1093/ismejo/wrag232 (PMC13592775; doi:10.1093/ismejo/wrag232)
Supplement: Supplementary file 1 [file 1_supplement_revised_clean_wrag232.pdf]

Supplementary material for
Lifestyle Differentiation Among Marine Denitrifying Microorganisms

Xin Sun[1,2*], Irene H. Zhang[3,4], Andrew R. Babbin[3], J. L. Weissman[5,6], Emily J. Zakem[2]

Contents include Supplementary Methods, Figures S1 – S3, Table S1, and references.

Supplementary Methods
Stoichiometry of microbial redox reactions

Microbial redox reactions were described in a previous modeling framework [1]. Briefly, $C_{6.6}H_{10.9}O_{2.6}N$ and $C_5H_7O_2N$ represent compositions of OM and biomass (B), respectively. d (d_{OM} and d_B) is defined to normalize redox reactions to one electron. To achieve mass balance in the equations:

$$d_{OM} = 4c_{OM} + h_{OM} - 2o_{OM} - 3n_{OM}$$
$$d_B = 4c_B + h_B - 2o_B - 3n_B$$

f is defined as the fraction of electrons used in biomass synthesis. H_2O and H^+ are ignored in the full reactions. The full reactions for each microbial functional type are listed below.

Aerobic heterotrophs, B_{het}

$$\frac{1}{d_{OM}}OM + \frac{1-f}{4}O_2 \rightarrow \frac{f}{d_B}B_{het} + \left(\frac{c_{OM}}{d_{OM}} - \frac{c_B f}{d_B}\right)CO_2 + \left(\frac{n_{OM}}{d_{OM}} - \frac{n_B f}{d_B}\right)NH_4^+$$

Denitrifiers that reduce NO_3^- to N_2, $B_{NO3-\rightarrow N2}$

$$\frac{1}{d_{OM}}OM + \frac{1-f}{5}NO_3^- \rightarrow \frac{f}{d_B}B_{NO3-\rightarrow N2} + \left(\frac{c_{OM}}{d_{OM}} - \frac{c_B f}{d_B}\right)CO_2 + \left(\frac{n_{OM}}{d_{OM}} - \frac{n_B f}{d_B}\right)NH_4^+ + \frac{1-f}{10}N_2$$

Denitrifiers that reduce NO_3^- to N_2O, $B_{NO3-\rightarrow N2O}$

$$\frac{1}{d_{OM}}OM + \frac{1-f}{4}NO_3^- \rightarrow \frac{f}{d_B}B_{NO3-\rightarrow N2O} + \left(\frac{c_{OM}}{d_{OM}} - \frac{c_B f}{d_B}\right)CO_2 + \left(\frac{n_{OM}}{d_{OM}} - \frac{n_B f}{d_B}\right)NH_4^+ + \frac{1-f}{8}N_2O$$

Denitrifiers that reduce NO_3^- to NO_2^-, $B_{NO3-\rightarrow NO2-}$

$$\frac{1}{d_{OM}}OM + \frac{1-f}{2}NO_3^- \rightarrow \frac{f}{d_B}B_{NO3-\rightarrow NO2-} + \left(\frac{c_{OM}}{d_{OM}} - \frac{c_B f}{d_B}\right)CO_2 + \left(\frac{n_{OM}}{d_{OM}} - \frac{n_B f}{d_B}\right)NH_4^+ + \frac{1-f}{2}NO_2^-$$

Denitrifiers that reduce NO_2^- to N_2, $B_{NO2-\rightarrow N2}$

$$\frac{1}{d_{OM}}OM + \frac{1-f}{3}NO_2^- \rightarrow \frac{f}{d_B}B_{NO2-\rightarrow N2} + \left(\frac{c_{OM}}{d_{OM}} - \frac{c_B f}{d_B}\right)CO_2 + \left(\frac{n_{OM}}{d_{OM}} - \frac{n_B f}{d_B}\right)NH_4^+ + \frac{1-f}{6}N_2$$

Denitrifiers that reduce NO_2^- to N_2O, $B_{NO2-\rightarrow N2O}$

$$\frac{1}{d_{OM}}OM + \frac{1-f}{2}NO_2^- \rightarrow \frac{f}{d_B}B_{NO2-\rightarrow N2O} + \left(\frac{c_{OM}}{d_{OM}} - \frac{c_B f}{d_B}\right)CO_2 + \left(\frac{n_{OM}}{d_{OM}} - \frac{n_B f}{d_B}\right)NH_4^+ + \frac{1-f}{4}N_2O$$

Denitrifiers that reduce N_2O to N_2, $B_{N2O\rightarrow N2}$

$$\frac{1}{d_{OM}}OM + \frac{1-f}{2}N_2O \rightarrow \frac{f}{d_B}B_{N2O\rightarrow N2} + \left(\frac{c_{OM}}{d_{OM}} - \frac{c_B f}{d_B}\right)CO_2 + \left(\frac{n_{OM}}{d_{OM}} - \frac{n_B f}{d_B}\right)NH_4^+ + \frac{1-f}{2}N_2$$

Parameterization for copiotrophic types

The denitrifier functional groups in the original modeling framework [1] are defined as oligotrophic types in this study. We add one copiotrophic type for each denitrifier functional group. For nitrogen, the

maximum uptake rate (V_{max}) of the copiotrophic type doubles the V_{max} of the oligotrophic type and the half saturation constant (K) of the copiotrophic type is ten times that of oligotrophs, following a previous study [2]. For OM, the K value of the copiotrophic type is twenty times that of oligotrophs to ensure that all $R*_{OM}$ values of copiotrophs are larger than those of oligotrophs (Fig. S1). V_{max} for OM of the copiotrophic type is four times that of oligotrophs, so that copiotrophs have lower affinity, defined as $\frac{Vmax}{K}$, and that the ratio of copiotrophic to oligotrophic $\frac{Vmax}{K}$ (= 0.2) is the same for OM and nitrogen. The steady state outcomes, including which microbes persist, are determined by their relative $R*$ values. Thus, alternative parameter combinations that yield the same relationship among $R*$ would be expected to produce the same qualitative outcomes.

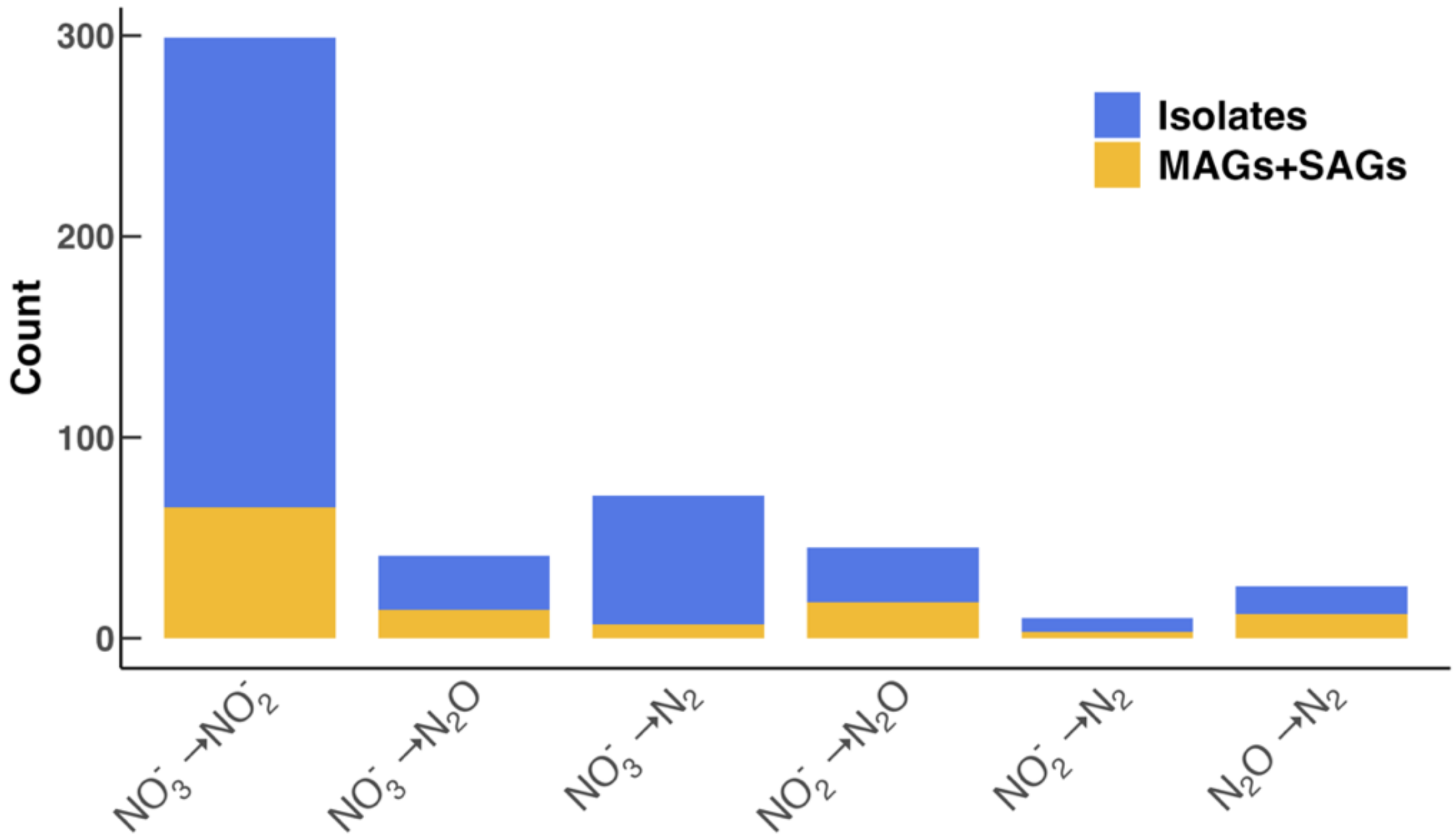

Figure S1 Counts of (draft) genomes in each functional group in our marine denitrifier dataset.

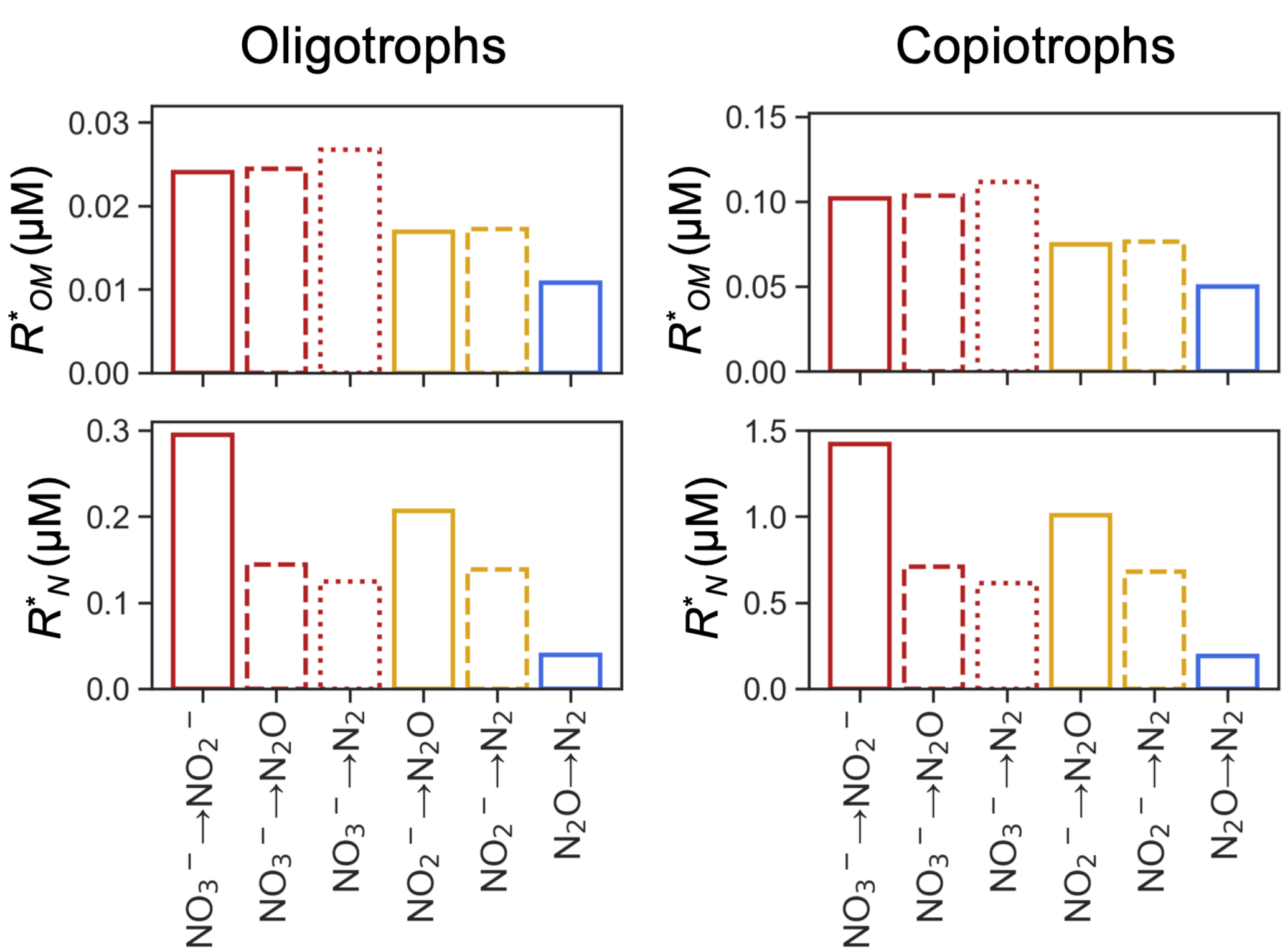

Figure S2 R^*_{OM} and R^*_N values for copiotrophic and oligotrophic lifestyles.

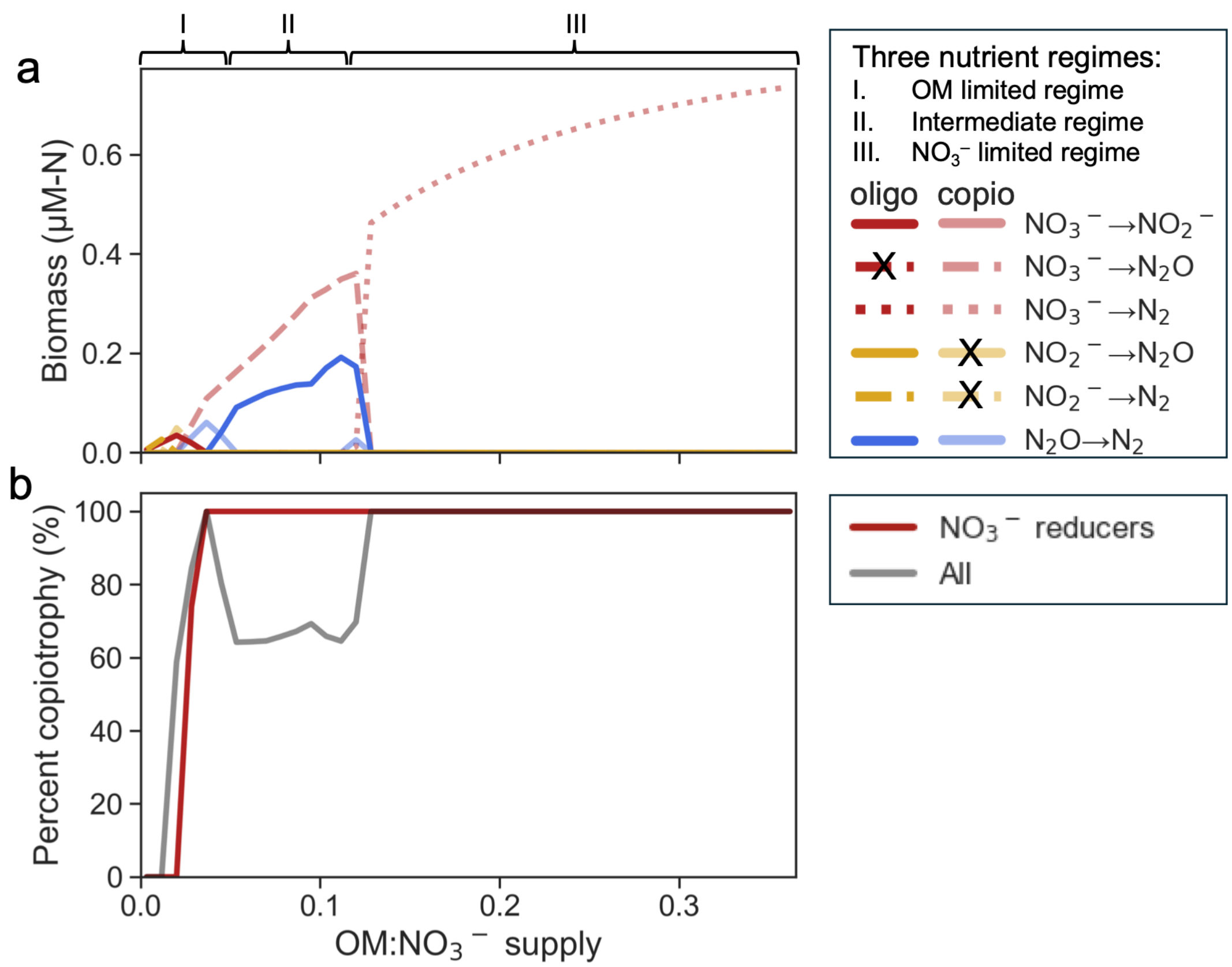

Figure S3 Resulting modeled biomasses and percent copiotrophy of different denitrifiers along an OM:NO_3^- supply gradient under the alternative assumption that copiotrophic and oligotrophic strategies differ only in organic matter acquisition, while nitrogen acquisition traits remain identical.

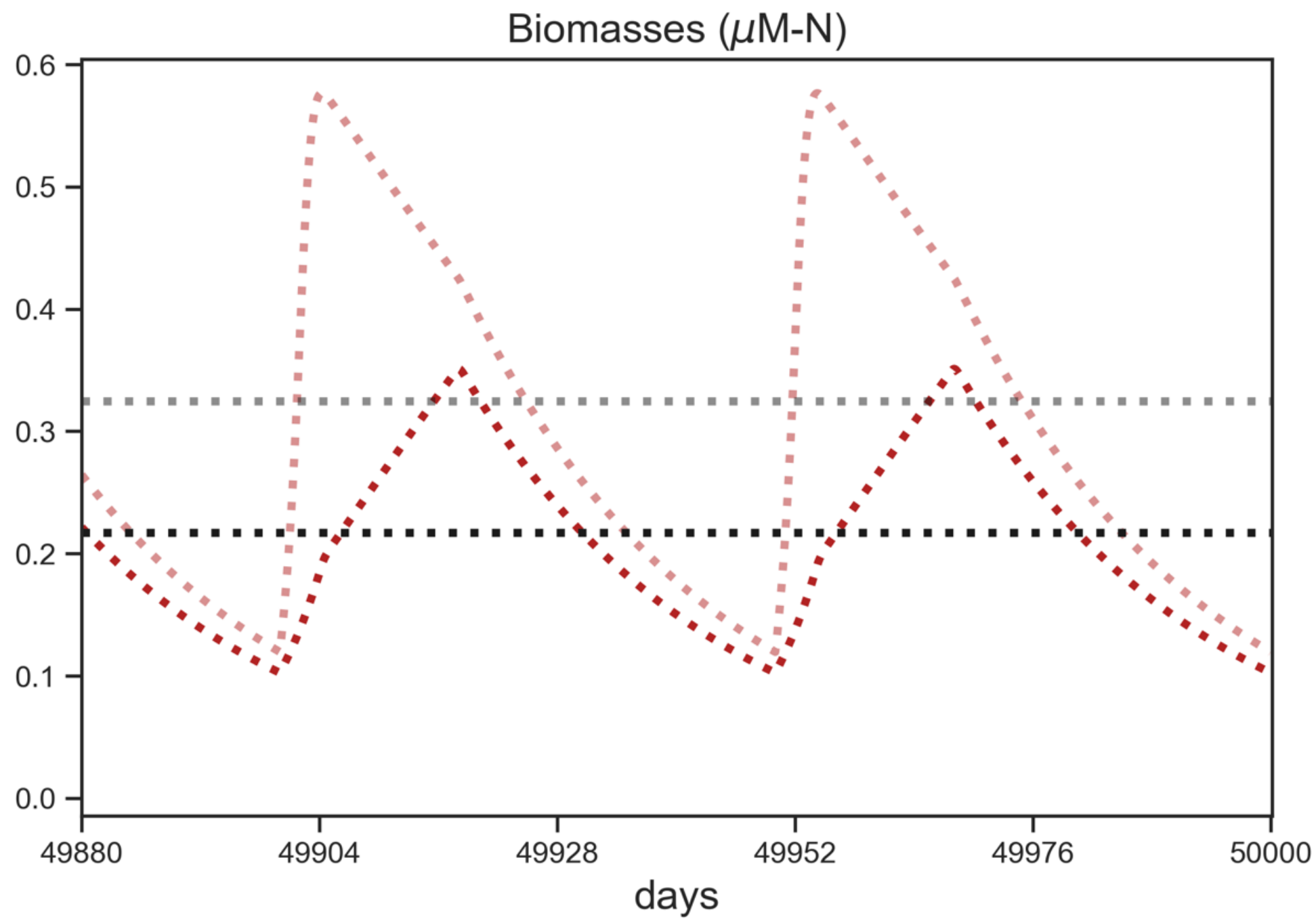

Figure S4 Example time series illustrating the quasi-equilibrium (limit-cycle) state reached by the model at an OM:NO_3^- supply ratio of 0.17. Dotted lines indicate $NO_3^- \rightarrow N_2$ denitrifier functional types. The more transparent lines indicate copiotrophs, while the less transparent lines indicate oligotrophs. Dark red lines are biomass oscillating at the quasi-equilibrium state, and black lines are averaged biomass over time.

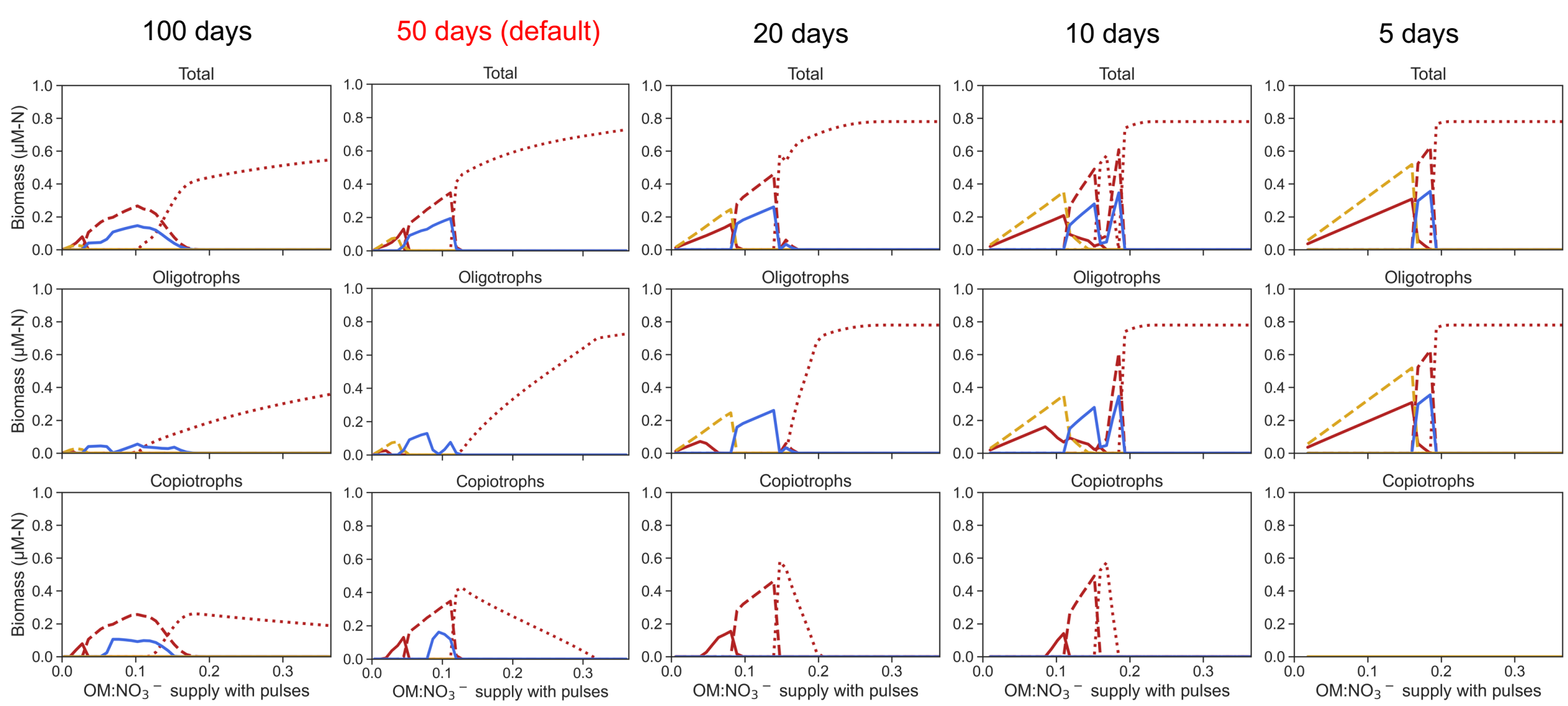

Figure S5 Effect of OM pulse frequency on the niches of denitrifier types.

Table S1 Sampling information for metagenomes used for relative abundance estimation in this study. Sampling and sequencing methods, read quality, and data availability for these samples are described in a previous study [3].

OMZ	Cruise	Station	Latitude	Longitude	Depth (m)	Oxygen (μM)
ETNP	RB-16-03	PS6	18.7	-104.4	53	16.4
ETNP	RB-16-03	PS6	18.7	-104.4	120	1.1
ETNP	RB-16-03	PS6	18.7	-104.4	200	1.2
ETNP	RB-16-03	14	17.2	-110.7	10	201
ETNP	RB-16-03	14	17.2	-110.7	185	0.03
ETNP	RB-16-03	14	17.2	-110.7	215	0.02
Arabian Sea	KNOX009	2N	15.2	64.0	130	0.61
Arabian Sea	KNOX009	2N	15.2	64.0	150	0.63
Arabian Sea	KNOX009	2N	15.2	64.0	200	0.66
Arabian Sea	KNOX009	2N	15.2	64.0	400	0.84
ETNP	SR1805	PS2	16.0	-105.0	60	189
ETNP	SR1805	PS2	16.0	-105.0	95	0
ETNP	SR1805	PS2	16.0	-105.0	200	0
ETNP	SR1805	PS3	18.0	-102.0	16	120
ETNP	SR1805	PS3	18.0	-102.0	45	0
ETNP	SR1805	PS3	18.0	-102.0	800	0

References

1. Sun X et al. Ecological dynamics explain modular denitrification in the ocean. *Proc Natl Acad Sci U S A* 2024;**121**:e2417421121.
2. Zakem EJ, Polz MF, Follows MJ. Redox-informed models of global biogeochemical cycles. *Nat Commun* 2020;**11**:5680.
3. Zhang IH et al. Partitioning of the denitrification pathway and other nitrite metabolisms within global oxygen deficient zones. *ISME Commun* 2023;**3**:76.
